# ASCT2 (SLC1A5)-dependent glutamine uptake is involved in the progression of head and neck squamous cell carcinoma

**DOI:** 10.1038/s41416-019-0637-9

**Published:** 2019-12-10

**Authors:** Ze Zhang, Ruoyan Liu, Yanjie Shuai, Yuting Huang, Rui Jin, Xudong Wang, Jingtao Luo

**Affiliations:** 1 Department of Maxillofacial and Otorhinolaryngology Oncology and Department of Head and Neck Oncology, Tianjin Medical University Cancer Institute and Hospital, National Clinical Research Center for Cancer; Key Laboratory of Cancer Prevention and Therapy, Tianjin; Tianjin’s Clinical Research Center for Cancer, Tianjin, 300060 China; 2 Department of Gynecologic Oncology, Tianjin Medical University Cancer Institute and Hospital, National Clinical Research Center for Cancer; Key Laboratory of Cancer Prevention and Therapy, Tianjin; Tianjin’s Clinical Research Center for Cancer, Tianjin, 300060 China; 3 Cancer and Immunology, Children’s Research Institute, Children’s National Medical Center, Washington, DC 20010 USA

**Keywords:** Cancer metabolism, Head and neck cancer, Targeted therapies

## Abstract

**Background:**

Glutamine is an abundant and versatile nutrient in cancer cells. Head and neck squamous cell carcinoma (HNSCC) was reported to be dependent on mainly glucose, not glutamine, for producing the energy required for survival and proliferation.

**Methods:**

The roles of ASCT2 (SLC1A5) and associated glutamine metabolism were determined by the MTT, colony formation, glutamine uptake, intracellular glutathione, ROS detection, immunofluorescence, immunohistochemistry, and apoptosis enzyme-linked immunosorbent assays as well as animal studies.

**Results:**

We found that glutamine is also critical for HNSCC. In this study, ASCT2, an amino acid transporter responsible for glutamine transport, in addition to LAT1 and GLS, is overexpressed in HNSCC and associated with poor survival. Using both in vivo and in vitro models, we found that knocking down ASCT2 by shRNAs or miR-137 or the combination of silencing ASCT2 and pharmacologically inhibiting SNAT2 via a small-molecule antagonist called V-9302 significantly suppressed intracellular glutamine levels and downstream glutamine metabolism, including glutathione production; these effects attenuated growth and proliferation, increased apoptosis and autophagy, and increased oxidative stress and mTORC1 pathway suppression in HNSCC. Additionally, silencing ASCT2 improved the response to cetuximab in HNSCC.

**Conclusions:**

In summary, ASCT2-dependent glutamine uptake and subsequent glutamine metabolism are essential for HNSCC tumorigenesis, and the combination of glutamine uptake inhibitors and cetuximab presents a promising strategy for improving the outcomes of HNSCC patients.

## Background

Aberrant metabolism and metabolic reprogramming represent malignant tumour hallmarks that are required to sustain the high rate of proliferation of cancer cells, which have to compete for limited fuel in a crowded, nutrient-deprived microenvironment.^1,2^ Glutamine, an abundant and versatile nutrient, has received substantial attention, second only to glucose, for its important role in energy generation, amino acid production, nucleotide biosynthesis, redox homeostasis, and autophagy and signalling regulation in cancer cells.^3–5^ Glutamine not only serves as a critical source of carbon and nitrogen for macromolecular synthesis but also provides α-ketoglutarate (α-KG) for the tricarboxylic acid (TCA) cycle and suppresses reactive oxygen species (ROS) levels through glutathione production. These characteristics make targeting glutamine metabolism pharmacologically an appealing way to explore new clinical strategies for treating cancer.

The cells in most tissues can synthesise glutamine. However, due to the rapid growth of tumours, the demand for glutamine far exceeds its supply, and the uptake of glutamine from the extracellular environment becomes essential.^3^ ASCT2, a Na +-dependent neutral amino acid transporter encoded by solute-linked carrier family A1 member 5 (SLC1A5), mediates the exchange of amino acid substrates and accounts for glutamine transport.^6^ ASCT2 is associated with tumour progression in multiple tumour types, such as prostate cancer, breast cancer, gastric cancer, endometrial cancer, and lung cancer.^7–11^ Nevertheless, the potential roles of glutamine and ASCT2 in different tumour types could be distinct due to various tissue origins and the tumour microenvironment.^3,12^ A few studies have found that glutaminolysis is involved in head and neck squamous cell carcinoma (HNSCC).^13,14^ The function of glutamine transporters HNSCC progression remains largely uncertain. A previous immunohistochemical study found that the expression of ASCT2 is significantly associated with advanced T stage, lymph node metastasis and lymphatic permeation in tongue cancer.^15^ Based on the role of ASCT2 in other cancers and the relationship between ASCT2 and clinicopathological features in tongue cancer, we hypothesise that ASCT2 transports glutamine into HNSCC cells and that glutamine metabolism subsequently supports cancer cell growth and survival.

In this study, we found that silencing ASCT2 significantly decreased intracellular glutamine and glutathione levels, thereby attenuating the growth of HNSCC both in vitro and in vivo. ASCT2 was also found to be required for the inhibition of apoptosis and autophagy in HNSCC. In addition, ASCT2-deficient cancer cells presented a better response to cetuximab, an FDA-approved therapeutic antibody targeting EGFR. Recently, H. Charles Manning’s team developed a small-molecule antagonist named V-9302, which was first reported as an ASCT2 inhibitor.^16^ However, the functional mechanism of V-9302 has been challenged, with V-9302 being shown to actually target SNAT2(SLC38A2) and not ASCT2.^17^ It was recently reported that depletion of ASCT2 leads to the induction of two other transporters, SNAT1 and SNAT2, which is sufficient to compensate for glutamine uptake.^18,19^ Therefore, the combination of silencing ASCT2 with V-9302 is a promising method to further decrease intracellular glutamine levels. We also explored the efficacy of using this new strategy to inhibit glutamine transport in HNSCC.

## Methods

### Patients

Tumour specimens from patients treated at the Department of Head and Neck Surgical Oncology, Tianjin Medical University Cancer Institute & Hospital, Tianjin, China during 2008–2012 were used for immunohistochemical evaluation of the relationship between ASCT2, LAT1, GLS or GLS2 expression and survival results as well as the relationship between mTOR activation (phosphorylated mTOR) and ASCT2. Tumour specimens from 86 patients who underwent post cetuximab surgery were used for immunohistochemical evaluation of the relationship between ASCT2 expression and response to cetuximab. The study was conducted with approval from the ethics committee of Tianjin Cancer Hospital, and informed consent was obtained for experimentation.

### Cell culture

Human HNSCC cells (SCC15 and FaDu) were maintained in Dulbecco’s modified Eagle’s medium/F12 medium supplemented with 10% foetal bovine serum, 2 mM glutamine, 100 units/ml penicillin, and 100 μg/ml streptomycin in a 5% CO_2_ atmosphere at 37 °C.

### Reagents

Cetuximab was provided by ImClone Systems, an Eli Lilly company. Dichloroacetate (DCA) was purchased from Sigma-Aldrich. V-9302 was purchased from AOBIOUS.

### Plasmids, shRNAs, and transfection

ASCT2 cDNA was amplified from SCC15 cells by RT-PCR. Primers were designed

based on a previous study as follows:^20^ forward primer, TAATACTAGTCACCATGGTGGCCGATCCTCCTCGAGACTCC; reverse primer, TAATGCGGCCGCACTTCCGTGATGGTGATGGTGATGCATGACTGATTCCTTCTCAG. ASCT2 cDNA was sub-cloned into the pcDNA3.1 expression vector via the *HindIII* and *XbaI* sites. ASCT2-targeted shRNAs (#1, CCGGGCCTGAGTTGATACAAGTGAACTCGAGTTCACTTGTATCAACTCAGGCTTTTTG; #2, CCGGGCCTGAGTTGATACAAGTGAACTCGAGTTCACTTGTATCAACTCAGGCTTTTTG) and control shRNA were purchased from Sigma-Aldrich. The miR-137 overexpression cDNA was designed according to a previous study as follows:^21^ forward primer, GCTCAGCGAGCAGCAAGAGT; reverse primer, GGCAATAAGAGCGAAACACCA. All constructs were verified by sequence analysis (GENEWIZ, Beijing, China).

To generate stable cell lines expressing shRNAs or cDNAs, HEK293T cells were transfected with a lentivirus-specific expression vector or scramble vector and packaging plasmid mix using Lipofectamine 3000 transfection reagent (Invitrogen, USA). Forty-eight hours after transfection, the supernatant containing viruses was collected and used to infect HNSCC cells with 8 μg/ml polybrene. Then, 2 µg/ml puromycin (Sigma-Aldrich, USA) was used to select infected cells for one week. The efficiency of silencing or overexpression was assessed by western blot.

### Western blotting

Cells were harvested and lysed in lysis buffer for 30 min at 4 °C, and total protein was quantified using a BCA protein assay kit (Thermo Fisher Scientific, USA). The proteins were dissociated and separated by SDS/PAGE and then transferred to polyvinylidene difluoride (PVDF) membranes, which were incubated with primary antibodies. The primary antibodies used for western blotting and their sources were as follows: anti-ASCT2 (Cell Signaling Technology #8057), anti-PARP (Cell Signaling Technology #9532), anti-LC3B (Cell Signaling Technology #3868), anti-phosphorylated p70S6K (Thr421/Ser424) (Cell Signaling Technology #9204), anti-p70S6K (Cell Signaling Technology #2708), anti-phosphorylated S6 (Ser235/236) (Cell Signaling Technology #4858), anti-S6 (Cell Signaling Technology #2317) and anti-β-actin (Cell Signaling Technology #3700). Antigen-antibody complexes were detected using horseradish peroxidase-conjugated secondary antibodies (Cell Signaling Technology #7074; #7076) with enhanced chemiluminescence (ECL) western blot detection reagent (Merck Millipore).

### Glutamine uptake and intracellular glutathione assays

The glutamine uptake assay was performed according to the procedure described in a previous study.^22^ In brief, after digestion with trypsin, the cells were resuspended in glutamine-deficient medium containing ^3^H-labelled glutamine (Perkin Elmer). After incubation for 5 min at 37 °C, the cells were washed with cold PBS. Then, the cells were lysed with 0.2% SDS/0.2 N NaOH solution and incubated for 60 min. After neutralisation with 1 N HCL, the relative glutamine uptake was analysed with a scintillation counter.

Intracellular glutathione assays were performed using a glutathione assay kit (Cayman Chemical). After the cells were collected by centrifugation (2000 × *g* for 10 min at 4 °C), they were resuspended in 500 μl of cold buffer (50 mM MES buffer (pH 6–7) containing 1 mM EDTA) and sonicated. Then, the supernatant was removed after centrifugation at 13,000 rpm for 15 min at 4 °C and stored on ice. The supernatant was deproteinated by precipitation with 10% metaphosphoric acid and centrifuged at 5000 rpm for 5 min. The cleared supernatant was neutralised with triethanolamine. An aliquot of each sample was transferred to a 96-well microplate well to detect total glutathione according to the manufacturer’s instructions. This detection was based on the reaction catalysed by glutathione reductase to convert oxidised glutathione (GSSG) to GSH; the yellow product 5-thio-2-nitrobenzoic acid (TNB) was produced after the reaction of the sulfhydryl group of GSH with 5,5′-dithio-bis-2-nitrobenzoic acid (DTNB), which was quantified at 405 nm using spectrophotometry.

### ROS detection

An intracellular ROS detection assay was performed using a total ROS detection kit (Enzo Life Sciences). Briefly, after the indicated treatment, cells were stained with a ROS detection solution for 60 min at 37 °C in the dark, and the ROS detection mix was then removed from the glass slides. After carefully washing with wash buffer twice, the cells were observed by a fluorescence microscope using standard excitation/emission filter sets (ex/em: 490/525 nm).

### Cell survival and proliferation assays

Cell survival and proliferation assays were performed using the methyl-thiazolyl diphenyl-tetrazolium bromide (MTT) method. After seeding cells into 96-well plates, a total volume of 20 μl of MTT (Solarbio, China, 5 mg/ml) was added for 4 h at 37 °C in the dark. After removal of the medium and MTT, 200 μl of DMSO was added to each well, and the optical density (OD) at an absorbance wavelength of 490 nm was measured by spectrophotometry.

### Colony formation assay

A total of 5 × 10^2^ cells were grown in 60-mm plates containing complete growth medium for 14 days. Then, the cells were fixed with 4% paraformaldehyde (Sigma-Aldrich, USA) and stained with crystal violet (Zhangshan, China). The number of colonies was determined by ImageJ software. Colony formation assays were repeated in triplicate.

### Animal studies

Swiss female nude mice (5–6 weeks old) purchased from Taconic Biosciences (USA) were divided randomly into groups (five mice per group), and their right flanks were subcutaneously injected with cells infected with a pLEX-based recombinant lentivirus containing firefly luciferase cDNA. For the tumour growth assay, infected cells from stable single cell clones of shASCT2 and control cells (6 × 10^6^ cells in 100 μl of serum-free DMEM) were used for each nude mouse. When the xenograft volume reached approximately 120 mm^3^, V-9302 (75 mg per kg of body weight, daily) or vehicle treatment was begun. V-9302 was reconstituted in a vehicle of PBS supplemented with 2% DMSO and administered intraperitoneally as described before^16^ in the animal laboratory. For the cetuximab sensitivity assay, treatment with vehicle or cetuximab (250 mg/mouse twice a week) was started 9 days after tumour injection for FaDu cells and 6 days after injection for SCC15 cells when the xenograft volume reached approximately 100 mm^3^ in the animal laboratory. Bioluminescence imaging for tumour volume was collected each week under ether anaesthesia using an IVIS Lumina Imaging System (Caliper Life Sciences, USA) and D-Luciferin firefly potassium salt (Caliper Life Sciences, USA), and body weight was measured daily. After 35 days, the animals were sacrificed by cervical dislocation under ether anaesthesia and the tumours were collected for further pathological examination. The National Institutes of Health guide for the care and use of laboratory animals was followed. All animal study protocols were also approved by the Institutional Animal Care and Use Committee of Tianjin Medical University Cancer Institute and Hospital.

### Immunohistochemistry analysis

Paraffin-embedded tissue samples were cut into 4-μm-thick sections. After baking for 2 h (68 °C), pathological sections were deparaffinised in xylene and rehydrated in graded ethanol solutions. The sections were boiled with citrate buffer under high pressure for 3 min for antigenic retrieval and then incubated with one of the following primary antibodies overnight at 4 °C: anti-ASCT2 (1:400; Cell Signaling Technology #8057), anti-LAT1 (1:50; Proteintech 13752-1-AP), anti-GLS (1:50; Proteintech), anti-GLS2 (1:800 Abcam ab113509) and anti-p-mTOR (1:100; Cell Signaling Technology #2976). After washing, a secondary antibody (Zhongshan Biotech, Beijing, China) was used. The sections were incubated with DAB (3,3-diaminobenzidine), counterstained, dehydrated and mounted in permanent mounting medium. Scoring was conducted according to the ratio and intensity of positively stained cells. The intensities were as follows: negative, 0; weakly positive, 1; moderately positive, 2; and strongly positive, 3. The estimated fraction of positively stained tumour cells was evaluated by percentage from 0 to 100%. The final score of protein expression was obtained by multiplying the intensity by the proportion score. All expression scores were performed in a blinded manner and determined independently by two pathologists. The patients were divided by the median value of the expression score.

### Immunofluorescence

HNSCC cells were fixed in 4% paraformaldehyde in PBS for 10 min, and permeabilisation buffer (0.5% Triton X-100 in PBS) was then used. LC3B was visualised with a primary antibody (1:100, Proteintech) at 4 °C for 16 h followed by incubation with a FITC-conjugated secondary antibody (1:200, Proteintech) at 37 °C for 30 min and DAPI for 10 min. Images were acquired with a fluorescence microscope (Zeiss).

### Apoptosis enzyme-linked immunosorbent assay (ELISA)

To quantitatively analyse the level of apoptosis, we used a Cell Death Detection ELISA kit (Roche Diagnostics Corp.) according to the manufacturer’s instructions. The test principle was as follows: the anti-histone antibody was fixed on the wall of the microplate module, and incubation buffer was used to saturate nonspecific binding sites. Then, nucleosomes in the sample were bound with the immobilised anti-histone antibody via their histone components. Anti-DNA-peroxidase (POD), which reacts with the DNA part of the nucleosome, was quantified based on the formation of an immunocomplex with ABTS (2,2′-azino-di-[3-ethylbenzthiazoline sulfonate^6^]). Thus, cytoplasmic histone-associated DNA fragments were quantitative.

### The Cancer Genome Atlas (TCGA) data collection

Gene expression data (500 cases) presented in fragments per kilobase million (FPKM) and the corresponding clinical information (528 cases) for HNSCC were downloaded from the official TCGA website (https://cancergenome.nih.gov/) on March 2017.

### Gene set enrichment analysis (GSEA)

GSEA was performed using GSEA 3.0 (http://www.broadinstitute.org/gsea/). Of the 500 HNSCC patients, the 125 with the highest ASCT2 expression and 125 with the lowest ASCT2 expression were divided into two groups. A nominal *p*-value < 0.05 and a false discovery rate (FDR) *q*-value < 0.25 were considered significant.

### Statistical analyses

Two groups were compared using Student’s *t*-test. Correlations between factors were assessed using the chi-square test and linear regression analysis. The Kaplan–Meier method and Cox proportional hazards model were used to evaluate prognostic results. R version 3.5.1 was used for statistical analyses.

## Results

### ASCT2, LAT1 and GLS expression was increased in HNSCC and inversely associated with patient survival

To investigate the role of glutamine metabolism in HNSCC, we first conducted an analysis evaluating the prognostic significance of two amino acid transporters, ASCT2 and L-type amino acid transporter 1 (LAT1), and two glutaminases, GLS and GLS2. As membrane transporters of amino acids, ASCT2 is the transporter for glutamine uptake, and LAT1 exchanges intracellular glutamine with other extracellular amino acids, such as leucine. The glutaminases GLS and GLS2 catalyse the conversion of glutamine to glutamate, which contributes to glutathione synthesis and can be converted to α-KG through glutamate dehydrogenases (GLUD1 or GLUD2) or aminotransferases.^3,23^

**Fig. 1:**
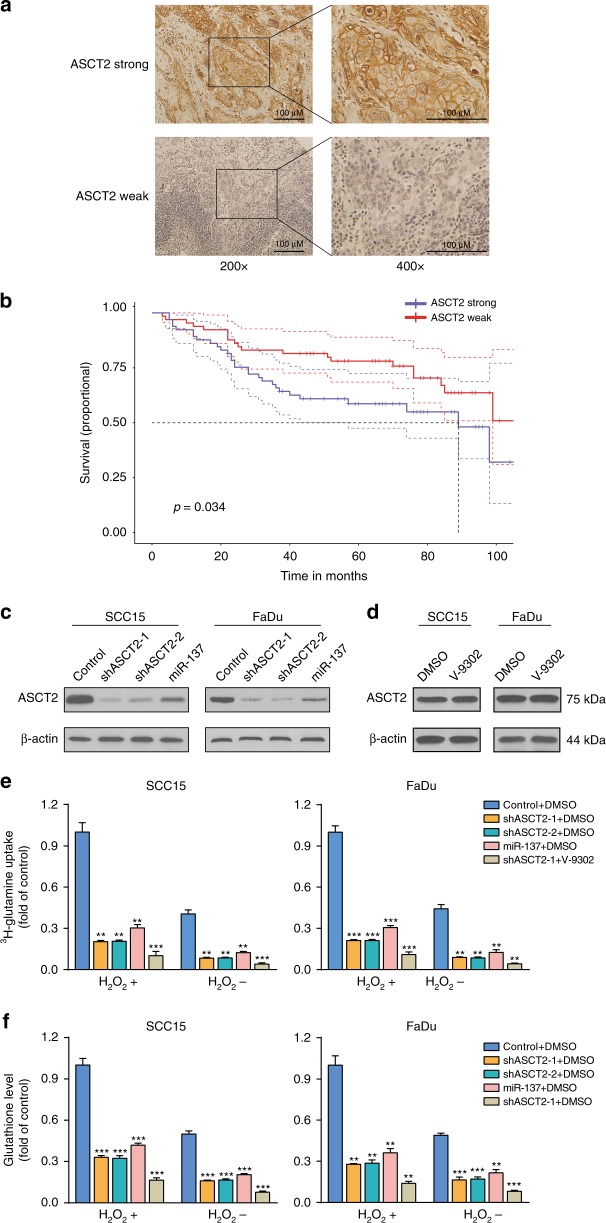
Subsection 1. The expression of ASCT2 is related to the survival of HNSCC patients. Representative images of immunohistochemical staining for ASCT2 in patients with HNSCC are shown (**a**). Magnifications of ×200 (left panels) and ×400 (right panels) are shown. Kaplan–Meier analysis results of the correlation of overall survival with ASCT2 expression are presented (**b**). Subsection 2. ASCT2 is required for glutamine uptake and glutathione biosynthesis. **c** Silencing ASCT2 in SCC15 and FaDu cells by transfection with two individual ASCT2 shRNAs or a miR-137 plasmid significantly decreased ASCT2 expression, as detected by western blotting. **d** V-9302 exposure did not influence the level of ASCT2 protein. **e** ^3^H-glutamine uptake was measured in SCC15 and FaDu cells with ASCT2 knockdown under V-9302 (25 µM, 48 h) or DMSO exposure and treatment with or without 1 mM H_2_O_2_. **f** Intracellular glutathione levels in SCC15 and FaDu cells treated with or without 1 mM H_2_O_2_ were measured using a Cayman glutathione assay kit. ‘*’, ‘**’, ‘***’ and ‘****’ represent ‘*p* < 0.05′, ‘*p* < 0.01′, ‘*p* < 0.001′ and ‘*p* < 0.0001′, respectively

Information from TCGA indicated that the mRNA levels of ASCT2, LAT1 and GLS are significantly higher in HNSCC tissues than in adjacent normal tissues (Fig. S1). However, GLS2 mRNA levels were not increased in HNSCC. Next, we assessed the protein levels of ASCT2, LAT1, GLS and GLS2 using immunohistochemistry and examined whether these protein expression levels are related to prognosis. A Kaplan–Meier analysis was performed to examine 124 HNSCC patients after surgical resection. The expression levels of ASCT2, LAT1 and GLS were significantly inversely associated with overall survival (Fig. 1a, b, Fig. S2A–D). A trend toward better overall survival with GLS2 expression was observed, although it was not significant (Fig. S2E–F). To further evaluate the relationship between ASCT2 expression and clinicopathological features, we analysed data from the HNSCC project of the TCGA and found that ASCT2 expression was significantly associated with sex and anatomic neoplasm subdivision. Notably, HPV-positive patients were likely to exhibit higher ASCT2 expression than HPV-negative patients. ASCT2 expression was also found to be associated with a worse differentiation grade and a higher probability of lymphovascular invasion (Table S1). We also explored the prognostic significance and expression of two other glutamine transporters, SNAT1 and SNAT2, using the TCGA data. The expression levels of both transporters were elevated, and SNAT2 expression was significantly associated with worse prognosis (Fig. S3).

### ASCT2 is required for glutamine uptake and glutathione biosynthesis

To evaluate the effect of ASCT2 on HNSCC glutamine uptake, we performed lentiviral transduction with a control shRNA or one of two different shRNAs (shASCT2-1 and shASCT2-2), and protein knockdown was verified in SCC15 and FaDu HNSCC cells by western blotting (Fig. 1c, d). miR-137 was recently reported to downregulate ASCT2 expression by directly targeting SLC1A5(ASCT2) mRNA.^21,24^ We verified that transfection of the miR-137 plasmid markedly reduced ASCT2 expression in both SCC15 and FaDu cells (Fig. 1c, d). As expected, a remarkable decrease in ^3^H-glutamine uptake was observed when ASCT2 was knocked down by the shRNAs or miR-137. The combination of shASCT2 with V-9302 further decreased glutamine uptake. As glutamine could increase the level of glutathione, a tripeptide that neutralises ROS, including H_2_O_2_, we investigated the effect of ROS on glutamine uptake in HNSCC cells. A significant increase in ^3^H-glutamine uptake was observed when H_2_O_2_ was added to the HNSCC cell culture. Knockdown of ASCT2 with or without V-9302 also decreased glutamine uptake upon the addition of ROS (Fig. 1e). Similarly, decreases in intracellular glutathione levels due to ASCT2 knockdown or the combination of shASCT2 with V-9302 were observed regardless of whether H_2_O_2_ was added (Fig. 1f).

### ASCT2 promotes HNSCC growth both in vitro and in vivo

**Fig. 2:**
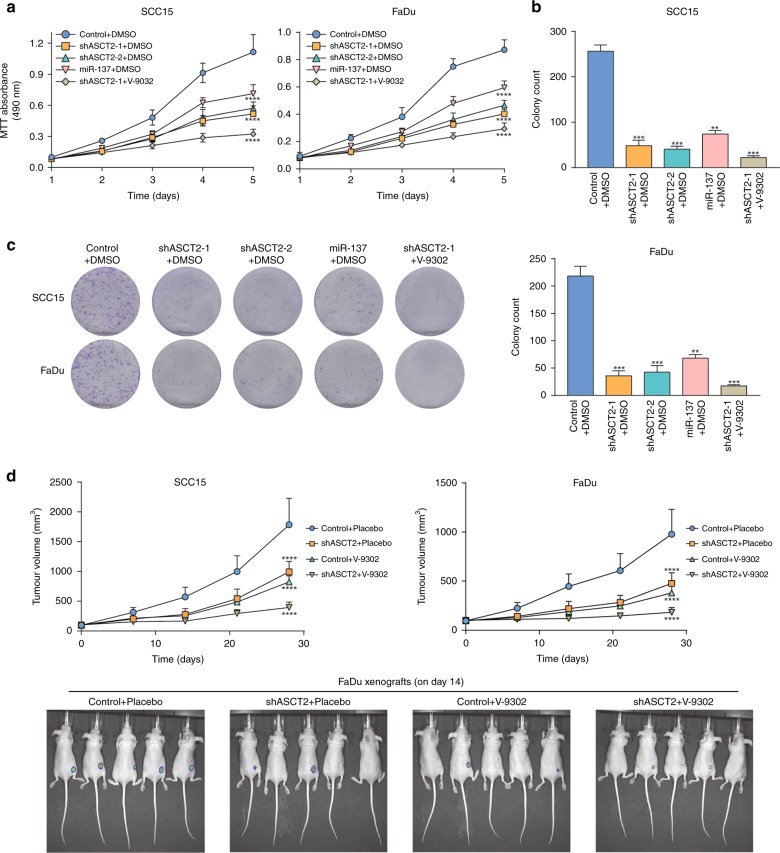
ASCT2 promotes HNSCC growth both in vitro and in vivo. **a** Proliferation of targeted ASCT2 cells with or without V-9302 (25 µM) exposure was evaluated by measuring cell viability by MTT assays. **b**, **c** Silencing ASCT2 or the combination of shASCT2 with V-9302 (25 µM) inhibited SCC15 and FaDu cell colony formation. After 14 days of incubation, the colonies were stained and counted by ImageJ software. **d** SCC15 or FaDu cells infected with a pLEX-based recombinant lentivirus containing firefly luciferase cDNA with and without targeted ASCT2 interference were implanted subcutaneously into the right flanks of nude mice (6 × 10^6^ cells/mouse in 100 ml of serum-free medium). Treatment with vehicle or V-9302 (75 mg per kg of body weight) started when the xenograft volume reached approximately 120 mm^3^. Images of the mice on day 14 (V-9302 or vehicle initiation as day 1) are shown. ‘*’, ‘**’, ‘***’ and ‘****’ represent ‘*p* < 0.05′, ‘*p* < 0.01′, ‘*p* < 0.001′ and ‘*p* < 0.0001′, respectively

To investigate the effect of ASCT2 and glutamine uptake on HNSCC growth, we performed an MTT assay. The cell growth curves showed that silencing ASCT2 or the combination of silencing ASCT2 and SNAT2 inhibition significantly attenuated the proliferation of both SCC15 and FaDu cells (Fig. 2a). Similarly, HNSCC cells transfected with the shRNAs or miR-137 exhibited suppressed colony formation, with fewer colonies (Fig. 2b). We further evaluated the effects of ASCT2 silencing and the strategy of silencing ASCT2 and treating with V-9302 together in the in vivo models. In accordance with the in vitro results, the subcutaneous xenografts of cells in which ASCT2 was knocked down exhibited a reduced growth tendency and a smaller tumour size than the control HNSCC cells. The combination of ASCT2 knockdown with V-9302 further suppressed the growth of the xenograft (Fig. 2c). Both the in vitro and in vivo findings indicated that targeting glutamine uptake suppressed the growth of HNSCC.

### ASCT2 inhibits apoptosis and autophagy in HNSCC

**Fig. 3:**
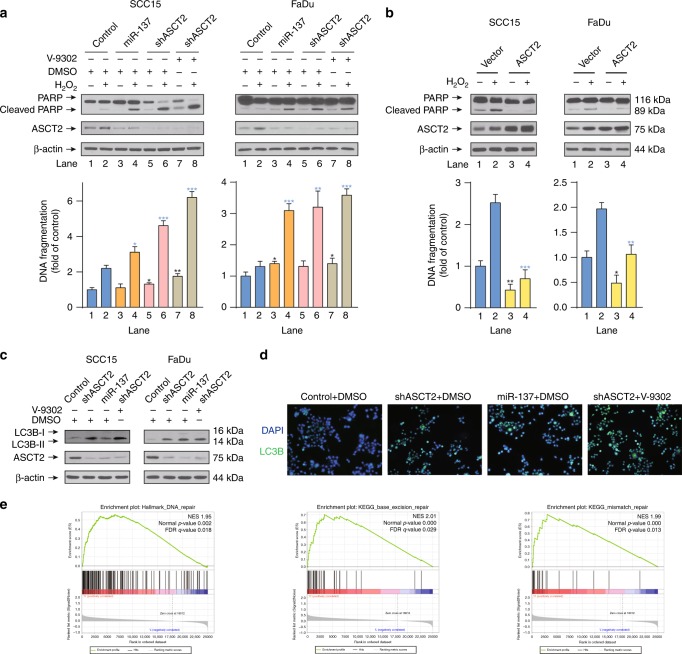
ASCT2 inhibits apoptosis and autophagy in HNSCC. **a** SCC15 and FaDu cells infected with a lentiviral vector expressing ASCT2 shRNA or a control vector were treated with either DMSO or V-9302 (25 µM, 48 h), followed by western blot detection of PARP cleavage and a quantitative apoptosis ELISA. **b** SCC15 and FaDu cells transfected with an ASCT2 construct or control vector and treated with or without 1 mM H_2_O_2_ for 24 h were lysed for the detection of PARP cleavage by western blotting. The results were also verified by a quantitative apoptosis ELISA. **c**, **d** SCC15 and FaDu cells transfected with ASCT2 shRNA or a control vector were exposed to DMSO or V-9302 (25 µM, 48 h). The expression of LC3B-II was evaluated by **c** western blotting, and LC3B-positive cells were detected by **d** immunofluorescence (images of SCC15 cells are shown). **e** Patients in the TCGA HNSCC project were divided into two groups (top 25% with high ASCT2 expression and bottom 25% with low ASCT2 expression). GSEA results show that ‘HALLMARK_DNA_REPAIR’, ‘KEGG_BASE_EXCISION_REPAIR’ and ‘KEGG_MISMATCH_REPAIR’ were differentially enriched in the high ASCT2 expression HNSCC patients. ‘*’, ‘**’, ‘***’ and ‘****’ represent ‘*p* < 0.05′, ‘*p* < 0.01′, ‘*p* < 0.001′ and ‘*p* < 0.0001′, respectively (‘*’ was coloured black with reference to the line 1, ‘*’ was coloured blue with reference to the line 2)

To investigate the role of ASCT2 in ROS-induced apoptosis in HNSCC cells, we treated SCC15 and FaDu cells with H_2_O_2_ to induce apoptosis. Successful apoptosis induction was shown by the appearance of PARP cleavage and via a quantitative apoptosis ELISA. Targeting ASCT2 with the shRNAs or the combination of shASCT2 with V-9302 sensitised both SCC15 and FaDu cells to a fairly low dose of H_2_O_2_ (0.1 mM), as evidenced by obvious PARP cleavage (Fig. 3a). In contrast, ASCT2 overexpression protected HNSCC cells from apoptosis after a high dose of H_2_O_2_ (1 mM) (Fig. 3b). The results were verified by a quantitative apoptosis ELISA (Fig. 3a, b). We also explored the role of ASCT2 in autophagy in HNSCC cells. Western blot results showed increased expression of the microtubule-associated protein 1 light chain 3B (LC3B)-II, a marker of autophagy, upon silencing ASCT2 with or without V-9302 exposure in HNSCC cells (Fig. 3c). This finding was further confirmed by immunofluorescence assays in which similar results were obtained (Fig. 3d). Interestingly, GSEA of patients with the top and bottom 25% SLC1A5 (ASCT2) expression levels in the TCGA HNSCC project showed significantly enriched gene sets of ‘HALLMARK_DNA_REPAIR’, ‘KEGG_BASE_EXCISION_REPAIR’ and ‘KEGG_MISMATCH_REPAIR’ in the high ASCT2 expression group, indicating that ASCT2 and the associated glutamine uptake may play an important role in DNA repair, which is critical for cell survival (Fig. 3e, Tables S2–3).

### ASCT2 promotes activation of the mTORC1 pathway

**Fig. 4:**
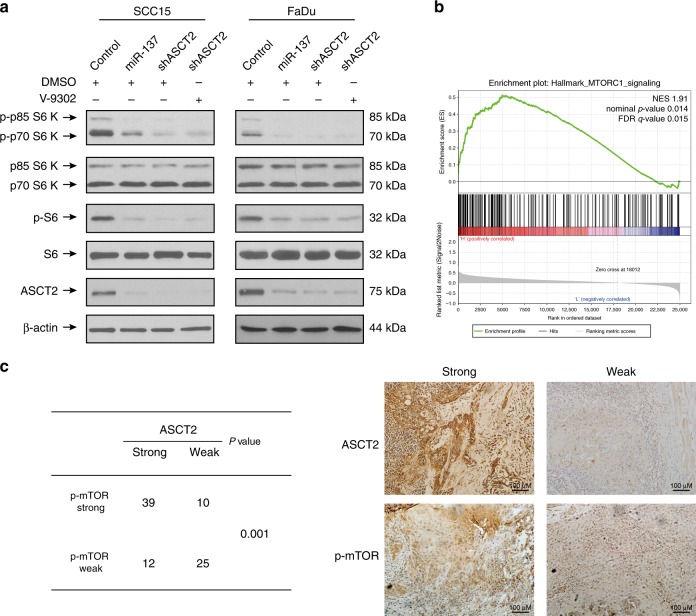
ASCT2 promotes the activation of the mTORC1 pathway. **a** SCC15 and FaDu cells infected with a lentiviral vector expressing ASCT2 shRNA or a control vector were treated with either DMSO or V-9302 (25 µM, 48 h), followed by the detection of phosphorylated p70S6K and phosphorylated S6 levels by western blotting. **b** Patients in the TCGA HNSCC project were divided into two groups (top 25% with high ASCT2 expression and bottom 25% with low ASCT2 expression). GSEA results show that ‘HALLMARK_MTORC1_SIGNALING’ genes were differentially enriched in high ASCT2 expression HNSCC patients. **c** HNSCC patients were divided into four groups according to ASCT2 and p-mTOR expression as measured by immunohistochemistry. The chi-square test was used to evaluate the relationship between ASCT2 and p-mTOR expression. Representative images of immunohistochemical staining are shown

Glutamine indirectly activates mechanistic target of rapamycin complex 1 (mTORC1), a central regulator of cell growth, mRNA translation, autophagy and metabolism.^5,25^ Furthermore, glutamine efflux drives the uptake of leucine by LAT1, and leucine is also required for mTORC1 activation. Thus, we hypothesised that targeting ASCT2 could inhibit the activation of the mTORC1 signalling pathway. As expected, silencing ASCT2 with or without V-9302 exposure in both SCC15 and FaDu (Fig. 4a) cells markedly decreased the phosphorylated p70S6K and phosphorylated S6 levels. Consistent with these data, GSEA showed the significantly enriched gene set of ‘HALLMARK_MTORC1_SIGNALING’ in the high ASCT2 expression group (Fig. 4b, Tables S2–3). Together, these findings indicate that ASCT2 promotes the activation of the mTORC1 pathway. We confirmed these results by analysing mTOR activity (phosphorylated mTOR expression) and ASCT2 expression using immunohistochemistry in human HNSCC tissues. The expression levels of p-mTOR and ASCT2 were positively correlated in HNSCC tissues (Fig. 4c).

### Silencing ASCT2 enhances ROS overproduction

**Fig. 5:**
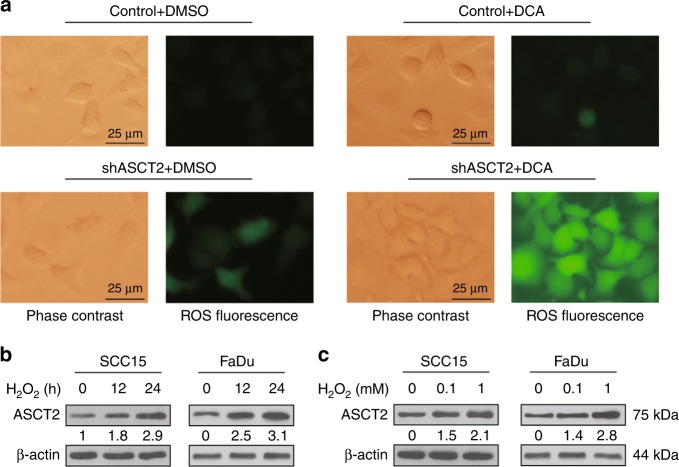
Silencing ASCT2 enhances DCA-induced ROS overproduction. **a** SCC15 and FaDu cells infected with a lentiviral vector expressing ASCT2 shRNA or a control vector were treated with either DMSO or DCA (10 mM, 48 h); then, the cells were analysed with an ROS detection kit and observed by a fluorescence microscope. Representative FaDu cells are shown. **b** SCC15 and FaDu cells treated with or without 1 mM H_2_O_2_ for 12 h or 24 h were lysed for the detection of ASCT2 by western blotting. **c** SCC15 and FaDu cells treated with or without 0.1 mM or 1 mM H_2_O_2_ for 24 h were lysed for the detection of ASCT2 by western blotting

Glutamine metabolism promotes glutathione synthesis, and the latter contributes to mitigating oxidative stress in proliferating cells as an intracellular antioxidant. Therefore, we speculate that targeting ASCT2 can increase the ROS level. A moderately elevated ROS level was observed in cells in which ASCT2 was knocked down compared to that in control HNSCC cells. Then, we treated SCC15 or FaDu cells with DCA, a chemical reported to induce ROS overproduction, mitochondrial depolarisation and apoptosis.^26^ Compared with that resulting from exposure to DCA alone, the combination of DCA and shASCT2 remarkably enhanced ROS overproduction (Fig. 5a). These results suggest that ASCT2 and the associated glutamine uptake indeed participate in antioxidant defence in HNSCC. We then explored whether ROS influenced the expression level of ASCT2. The addition of H_2_O_2_ increased ASCT2 protein expression in both a time- and dose-dependent manner (Fig. 5b, c).

### Targeting ASCT2 improved the response of HNSCC to cetuximab

**Fig. 6:**
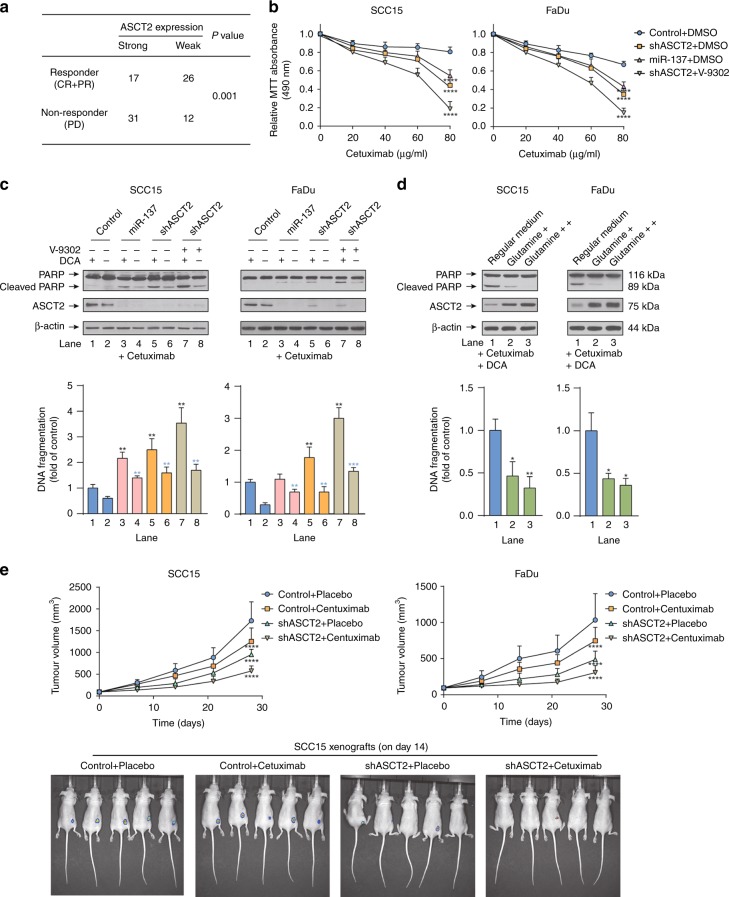
Targeting ASCT2 improved the response of HNSCC to cetuximab. **a** HNSCC patients treated with cetuximab were divided into two groups according to their ASCT2 expression as measured by immunohistochemistry. The chi-square test was used to evaluate the relationship between response to cetuximab and ASCT2 expression. The responders included complete responders (CRs) and partial responders (PRs), while non-responders were patients with progressive disease (PD). **b** The MTT assay showed that the inhibition of proliferation by cetuximab was enhanced by transfection with the two individual ASCT2 shRNAs or miR-137 or the combination of shASCT2 with V-9302 treatment (25 µM, 48 h). **c** SCC15 and FaDu cells infected with a lentiviral vector expressing ASCT2 shRNA or a control vector or treated with either DMSO or V-9302 (25 µM, 48 h) were treated with 20 nM cetuximab with or without 10 mM DCA. Cell lysates were subjected to western blotting for the detection of PARP cleavage. The results were verified by apoptosis ELISA. **d** The cells were treated with cetuximab plus DCA for 24 h in regular medium supplemented with additional glutamine. The results were verified by apoptosis ELISA. **e** SCC15 or FaDu cells were infected with a pLEX-based recombinant lentivirus containing firefly luciferase cDNA with and without targeted ASCT2 interference and then implanted subcutaneously into the right flanks of nude mice (6 × 100^6^ cells/mouse in 100 ml of serum-free medium). Treatment with vehicle or cetuximab (250 mg/mouse twice a week) started at 9 days after injection for FaDu cells and at 6 days after injection for SCC15 cells. Images of the mice on day 14 (cetuximab or vehicle initiation as day 1) are shown. ‘*’, ‘**’, ‘***’ and ‘****’ represent ‘*p* < 0.05′, ‘*p* < 0.01′, ‘*p* < 0.001′ and ‘*p* < 0.0001′, respectively (‘*’ was coloured black with reference to the line 1, ‘*’ was coloured blue with reference to the line 2)

Cetuximab, a recombinant monoclonal antibody that binds specifically to the extracellular domain of human EGFR, has been approved by the FDA for treating metastatic HNSCC.^27^ To evaluate whether silencing ASCT2 sensitises HNSCC to cetuximab, we first performed immunohistochemistry analysis to detect ASCT2 protein expression in the carcinoma tissues of HNSCC patients treated with cetuximab. ASCT2 expression in the carcinoma tissues was significantly higher in non-responders than in responders (Fig. 6a). The results of the MTT assay showed that the responses of SCC15 and FaDu cells to cetuximab were significantly improved with ASCT2 silencing with or without V-9302 exposure (Fig. 6b). The knockdown of ASCT2 and the combination of shASCT2 with V-9302, especially in combination with DCA, also sensitised SCC15 and FaDu cells to cetuximab-induced apoptosis (Fig. 6c). To confirm that a decrease in glutamine, rather than other amino acids, induced by targeting ASCT2 sensitised HNSCC cells to apoptosis, we cultured HNSCC cells with additional glutamine, and apoptosis induced by cetuximab and DCA was significantly attenuated (Fig. 6d). In vivo, HNSCC cell growth was inhibited by silencing ASCT2 or the addition of cetuximab alone compared with that in the control group, and the combination of cetuximab and shASCT2 remarkably suppressed SCC15 and FaDu xenografts (Fig. 6e). Together, these data indicate that targeting ASCT2 by shRNA transfection sensitised HNSCC to cetuximab both in vivo and in vitro.

## Discussion

Metabolic reprogramming is one of the most remarkable features of cancer cells. Since the first report nearly a century ago, no area has received more attention than aerobic glycolysis, a phenomenon termed ‘the Warburg effect’.^28^ However, recent work has indicated that fuels other than glucose also play an important role in the metabolic functions of cancer cells.^1^ Among the different types of nutrients, glutamine has attracted considerable attention because it is key to the survival, proliferation, differentiation state and stress resilience of cancer cells.^18^

A previous study found that HNSCC cells are mainly dependent on glucose, not glutamine, for producing the energy required for survival and proliferation.^29^ In contrast to this earlier study, our current work showed that glutamine uptake is also critical for proliferation, ROS homeostasis, apoptosis, autophagy, cell signalling and response to cetuximab in HNSCC. Indeed, previous studies also found that glutamine is involved in HNSCC. The results of a metabonomics study showed that glutaminase expression was higher in primary and metastatic HNSCC tissues, tumourspheres, and cancer stem cells than in control samples, and that exogenous glutamine induced stemness via glutaminase.^13^ Xin Li’ s team explored the strategy of targeting cellular glutamine metabolism to reduce HNSCC growth, which was suggested to be effective, especially in combination with metformin.^14^

In this study, we first investigated the correlation of survival with several glutamine-associated proteins and found that the expression of ASCT2, LAT1 and GLS was inversely associated with the survival of HNSCC patients. These findings indicated that glutamine could play a functional role in HNSCC development. LAT1, an amino acid transporter in cell membranes, acquires leucine from the extracellular environment and is dependent on intracellular glutamine; the obtained leucine then participates in metabolic processes, such as mTOR activation. Of the two glutaminase enzymes catalysing the conversion of glutamine to glutamate, GLS, not GLS2, was found to be associated with worse survival in HNSCC patients. The current finding is more logical when considering that GLS is frequently deregulated in cancer and silencing or inhibiting GLS decreases tumour growth in a number of models.^4,30,31^ Although the role of GLS2 in cancer remains unclear, GLS2 is a p53 target gene and seems to function in tumour suppression in some tissues.^32^ Considering the important function of glutamine in cancer cell proliferation and survival, restricting glutamine uptake presents an appealing method for treating cancer. ASCT2 is the transporter in charge of glutamine uptake. In our study, silencing ASCT2 expression significantly decreased glutamine uptake. However, the intracellular glutamine level was not extraordinarily low when ASCT2 was silenced alone, potentially because ASCT2 is not the only transporter for glutamine uptake. Two other glutamine transporters, SNAT1 and SNAT2, were induced to compensate for glutamine uptake.^18,19^ Therefore, targeting ASCT2, SNAT1 and SNAT2 together should be evaluated for their tumour suppression capability. In this study, we simultaneously silenced ASCT2 and treated HNSCC with V-9302, which further deceased glutamine uptake by inhibiting SNAT2. It was encouraging that the tumour inhibition effect achieved using these methods in combination was superior to that observed using either method alone.

By analysing data from a TCGA cohort, we found that HPV-positive patients are likely to exhibit higher ASCT2 expression than HPV-negative patients. Patients with HPV-negative and HPV-positive HNSCC have different socioeconomic profiles as well as clinical presentations and molecular profiles.^33^ Previous study has shown significant metabolic differences between HPV-negative and HPV-positive HNSCC, and HPV-negative tumours have a more glycolytic phenotype;^34^ thus, HPV-positive tumours possibly rely on other energy sources as compensation.

Transcription factors of the MYC family, proto-oncogenes that are deregulated in multiple cancers, including HNSCC, were reported to coordinate nutrient acquisition to produce energy and promote proliferation by triggering DNA replication and cell division. Evidence indicates that MYC directly drives glutamine uptake and catabolism by activating the expression of genes involved in glutamine metabolism, including ASCT2 and GLS.^4,35–37^ Recently, two independent studies reported that miR-137 suppresses glutamine metabolism by directly targeting SLC1A5 (ASCT2).^21,24^ We confirmed this mechanism in HNSCC. Notably, c-MYC was recently reported to directly suppress miR-137; thus, MYC regulates ASCT2 in dual mechanisms, collectively contributing to ASCT2 upregulation (just as GLS expression is upregulated by MYC both directly and indirectly through the MYC-mediated inhibition of miR-23 expression, which in turn suppresses GLS translation).^37^ In addition, in a previous study, miR-137 promoter methylation (which should promote ASCT2 expression) was associated with the poor survival of HNSCC patients, which is in line with our findings.^38^

In this study, we found that targeting ASCT2 suppresses HNSCC growth both in vitro and in vivo. Among the metabolism products that possibly act as limiting factors of proliferation, nucleotides and products of the TCA cycle, rather than ATP and NADPH, can be limiting for proliferation.^1^ On the one hand, as an important source of carbon, nitrogen from glutamine metabolism contributes directly to both purine and pyrimidine synthesis, which is critical for cell division and a common target of chemotherapy.^39^ Additionally, glutamine-dependent mTOR signalling activation and NADPH production could further support nucleotide biosynthesis.^3,40^ On the other hand, glutamine is converted to α-KG, an important TCA cycle intermediate, through glutaminases and glutamate dehydrogenases (GLUD1 or GLUD2), thereby replenishing the TCA cycle to provide precursors for several biomasses. Considering this information, it is easy to understand how silencing ASCT2 or the combination of ASCT2 with a SNAT2 inhibitor could delay HNSCC growth.

We also found that ASCT2-dependent glutamine metabolism could inhibit HNSCC cell death. A previous study found that cetuximab downregulates ASCT2 via cetuximab-mediated EGFR endocytosis, leading to enhanced ROS-induced apoptosis in HNSCC.^20,22^ In the present study, targeting ASCT2 alone was sufficient to sensitise HNSCC to ROS-induced apoptosis, which was potentially due to the decreased glutathione downstream of glutamine metabolism. Autophagy was also enhanced when ASCT2 was silenced. It is noteworthy that autophagy has a dual role in tumours, either supporting or suppressing tumour progression depending on the different tumour contexts.^5^ In stressful contexts, autophagy could promote the degradation of unnecessary and harmful cellular components and recycle nutrient sources, thus helping cancer cells survive in the crowded tumour microenvironment. Therefore, the simultaneous inhibition of both glutamine metabolism and autophagy could be a potential therapeutic strategy.^5^ However, the role of autophagy in HNSCC remains uncertain.^41^ Some studies also reported that autophagy activation promotes tumour cell death in HNSCC.^42,43^ Thus, autophagy modulation as a supplementary measure to glutamine uptake inhibition therapy in HNSCC should be used cautiously in the future.

Although pharmacological inhibition of ASCT2 appears to be a promising approach to treat cancers, it is hampered by the lack of specific inhibitors. L-γ-glutamyl-p-nitroanilide (GPNA) inhibits not only ASCT2 but also other amino acid transporters, such as LAT1, SNAT1 and SNAT2.^44,45^ A new compound called V-9302 was first reported to inhibit ASCT2 with a 100-fold improvement in potency compared with GPNA.^16^ However, Stefan Bröer’s research revealed that V-9302 does not inhibit ASCT2 but rather blocks SNAT2 and LAT1.^17^ Thus, a specific ASCT2 inhibitor remains to be identified thus far. As V-9302 was found to be sufficient to further decease glutamine uptake in ASCT2-depleted cells, the combination of silencing ASCT2 with V-9302 theoretically suppressed the tumour further, which was confirmed by our work. Significantly, as glutamine uptake is required not only in cancer cells but also in other healthy cells of the body, caution should be exercised in combining ASCT2 silencing with V-9302 treatment, which may result in toxic effects.

Targeting glutaminase was explored as a method to suppress glutamine metabolism and cancer cell growth.^1^ Compared with the strategy to inhibit GLS, there is reason to believe that blocking cellular glutamine uptake by the combination of silencing ASCT2 with V-9302 could be more beneficial than targeting downstream glutaminase activity, which does not fully inhibit the function of extramitochondrial glutamine. In addition, although EGFR is overexpressed in more than 90% of HNSCC, cetuximab and other EGFR inhibitors only modestly improved the results in recurrent or metastatic HNSCC, while severe so-called ‘financial toxicity’ was generated.^46,47^ Thus, it is clinically important to investigate potential strategies for improving the HNSCC response to cetuximab. Previous studies found that ASCT2 forms a heterotrimeric molecular complex with EGFR and AP1G1; thus, cetuximab could co-target ASCT2 via EGFR endocytosis and decrease intracellular glutamine levels in HNSCC.^20,22^ In contrast, the effect of ASCT2 targeting on the response of HNSCC to cetuximab remained unclear. Wangjun Liao’s team found that inhibition of ASCT2 increased the sensitivity of colorectal cancer to cetuximab.^48^ In HNSCC, our previous study, in which acetyl-CoA carboxylase (ACC) was found to rewire cancer metabolism to allow HNSCC cells to survive inhibition of the Warburg effect by cetuximab, provided a combination strategy of using an ACC inhibitor (such as 5-tetradecyloxy-2-furoic acid (TOFA)) and cetuximab to improve cetuximab therapy outcomes.^49^ In this study, we propose a new therapeutic strategy to increase the curative effect of cetuximab by simultaneously targeting glutamine uptake, which requires further preclinical validation.

In summary, our study indicates that the downregulation of ASCT2 by shRNA or miR-137 transfection with or without the inhibition of SNAT2 by the competitive small-molecule antagonist V-9302 significantly decreased the intracellular glutamine level, leading to attenuated growth and proliferation, increased apoptosis and autophagy, inactivation of the mTORC1 pathway, increased oxidative stress and an improved response to cetuximab in HNSCC (Fig. S4). These results indicated that the combination of glutamine uptake inhibition and cetuximab is an appealing regimen to refine and improve the outcomes of HNSCC patients.

## Supplementary information

Supplementary Materials

## Data Availability

The datasets generated and analysed during the current study are available from the corresponding author on reasonable request.
