# Supplementary material for: ASCT2 (SLC1A5)-dependent glutamine uptake is involved in the progression of head and neck squamous cell carcinoma
Source: Br J Cancer. 2019 Dec 10;122(1):82–93. doi: 10.1038/s41416-019-0637-9 (PMC6964701; doi:10.1038/s41416-019-0637-9)
Supplement: Supplementary file 1 — Supplementary Materials [file 41416_2019_637_MOESM1_ESM.docx]

**Supplementary Materials**

Supplementary Fig. 1 Proteins associated with glutamine uptake and metabolism are overexpressed in HNSCC. (A) ASCT2, (B) LAT1, (C) GLS and (D) GLS2 mRNA expression was determined in carcinoma tissues and adjacent tissues from the TCGA project.


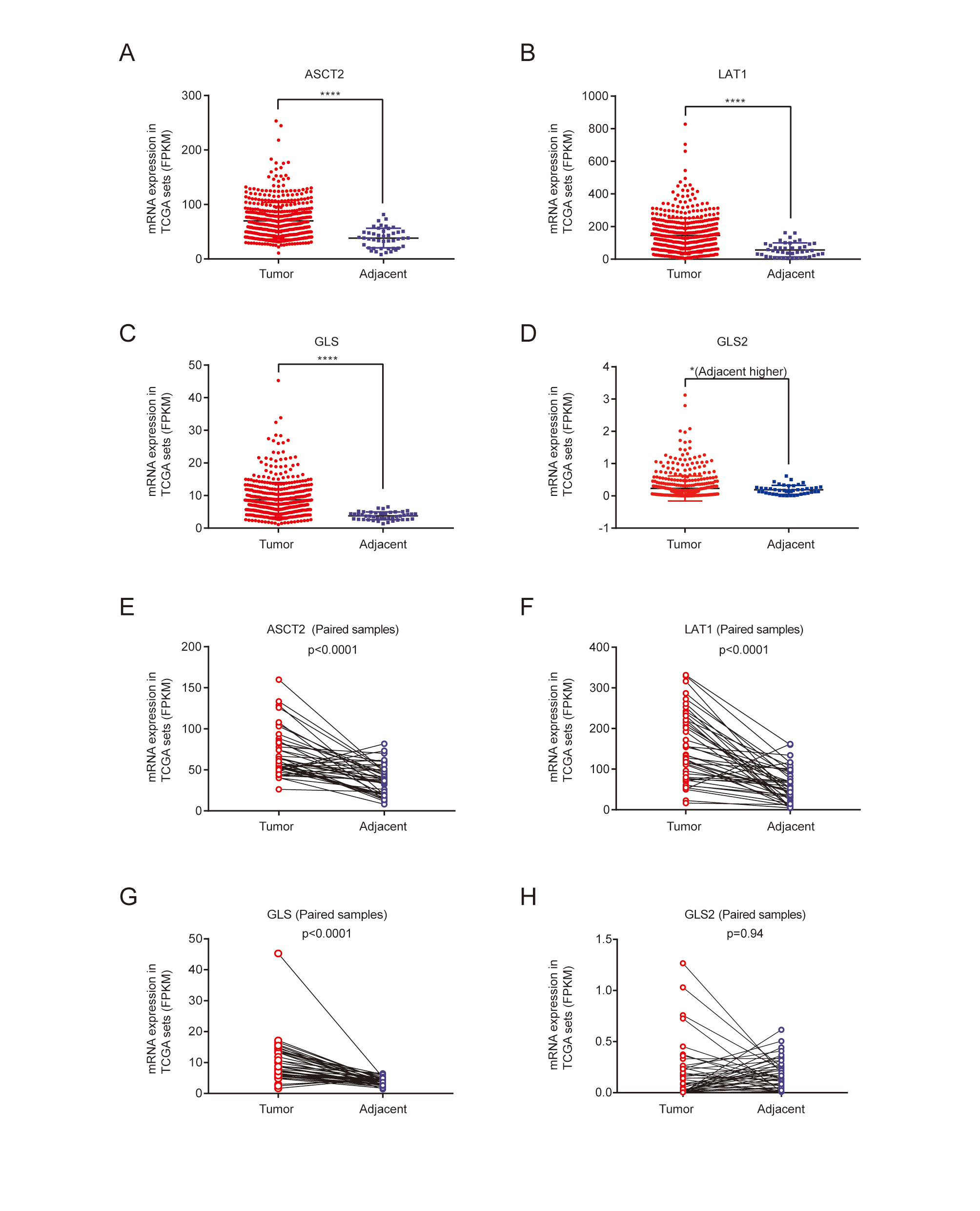


Supplementary Fig. 2 The expression of proteins associated with glutamine uptake and metabolism is related to the survival of HNSCC patients. Representative images of immunohistochemical staining for LAT1 (A), GLS (C) and GLS2 (E) in patients with HNSCC are shown. Magnifications of 200× (left panels) and 400× (right panels) are shown. Kaplan-Meier analysis results of the correlation of overall survival with LAT1 (B), GLS (D) and GLS2 (F) expression are shown.


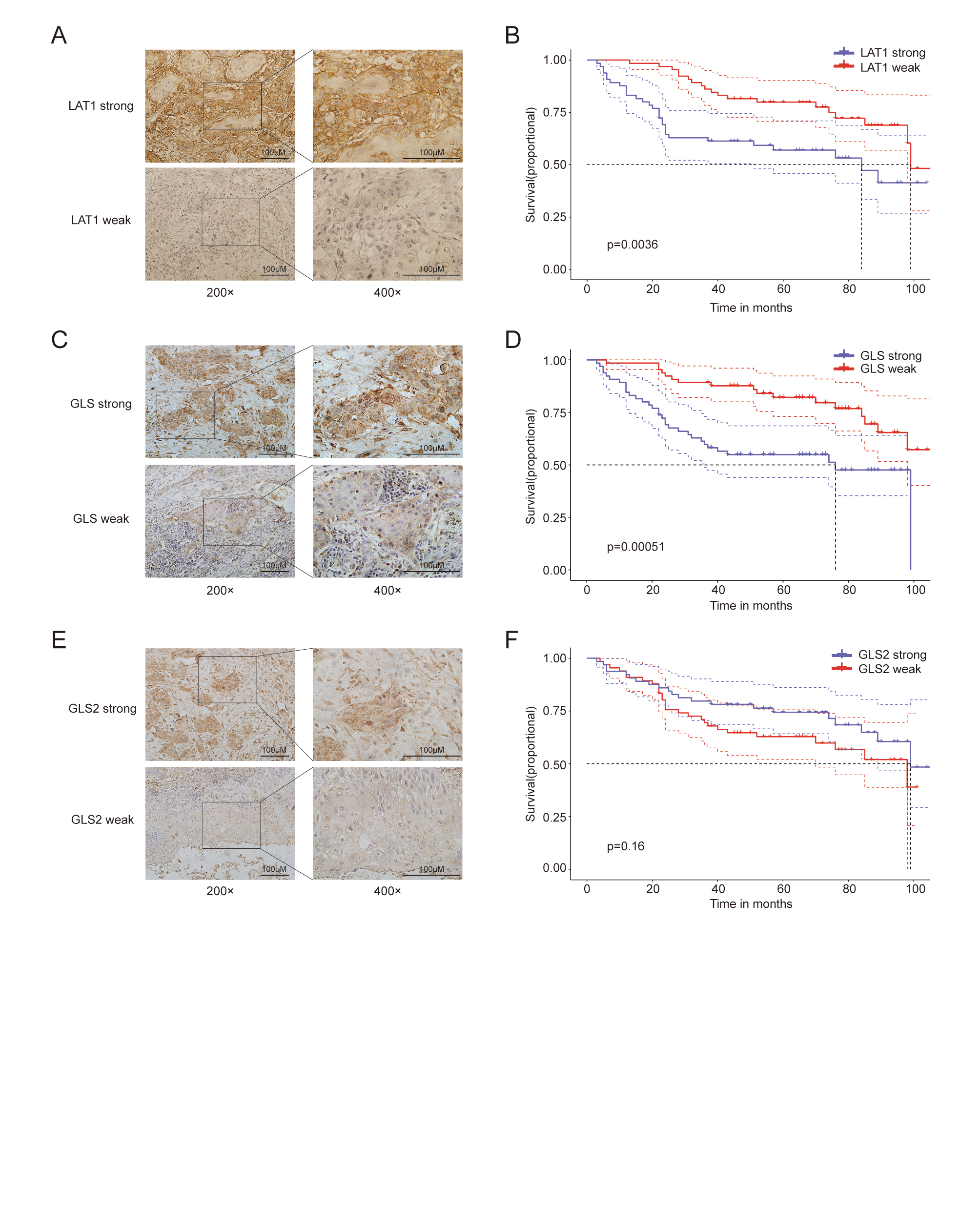


Supplementary Fig. 3 (A) SNAT1 and (B)SNAT2 are overexpressed in an HNSCC from the TCGA database. (C) SNAT1 was not significantly associated with prognosis, but (D)SNAT2 was significantly associated with worse prognosis in HNSCC.


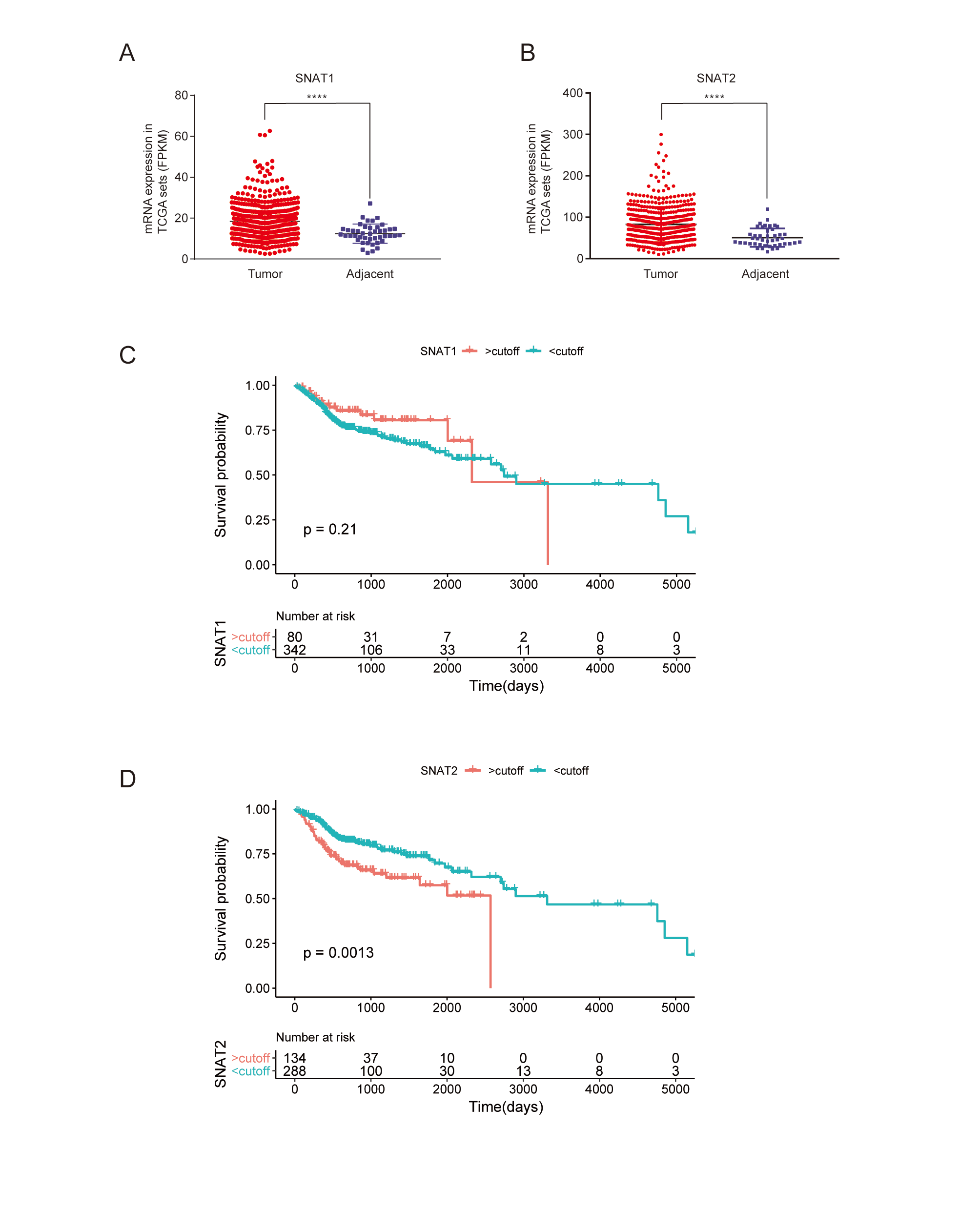


Supplementary Fig. 4 Proposed model of the mechanism underlying the effects of targeting ASCT2 on HNSCC. (A) In the wild situation, HNSCC acquires glutamine via ASCT2, and the subsequent glutamine metabolism functions in energy generation, amino acid production, nucleotide biosynthesis, redox homeostasis, autophagy regulation and signaling activation. (B) Several approaches, such as miR-137 transfection, suppress ASCT2-dependent glutamine uptake and metabolism, which can also be enhanced by inhibiting SNAT2 with V-9302, leading to attenuated growth and enhanced cell death in HNSCC. *Autophagy has a dual role in oncogenesis.


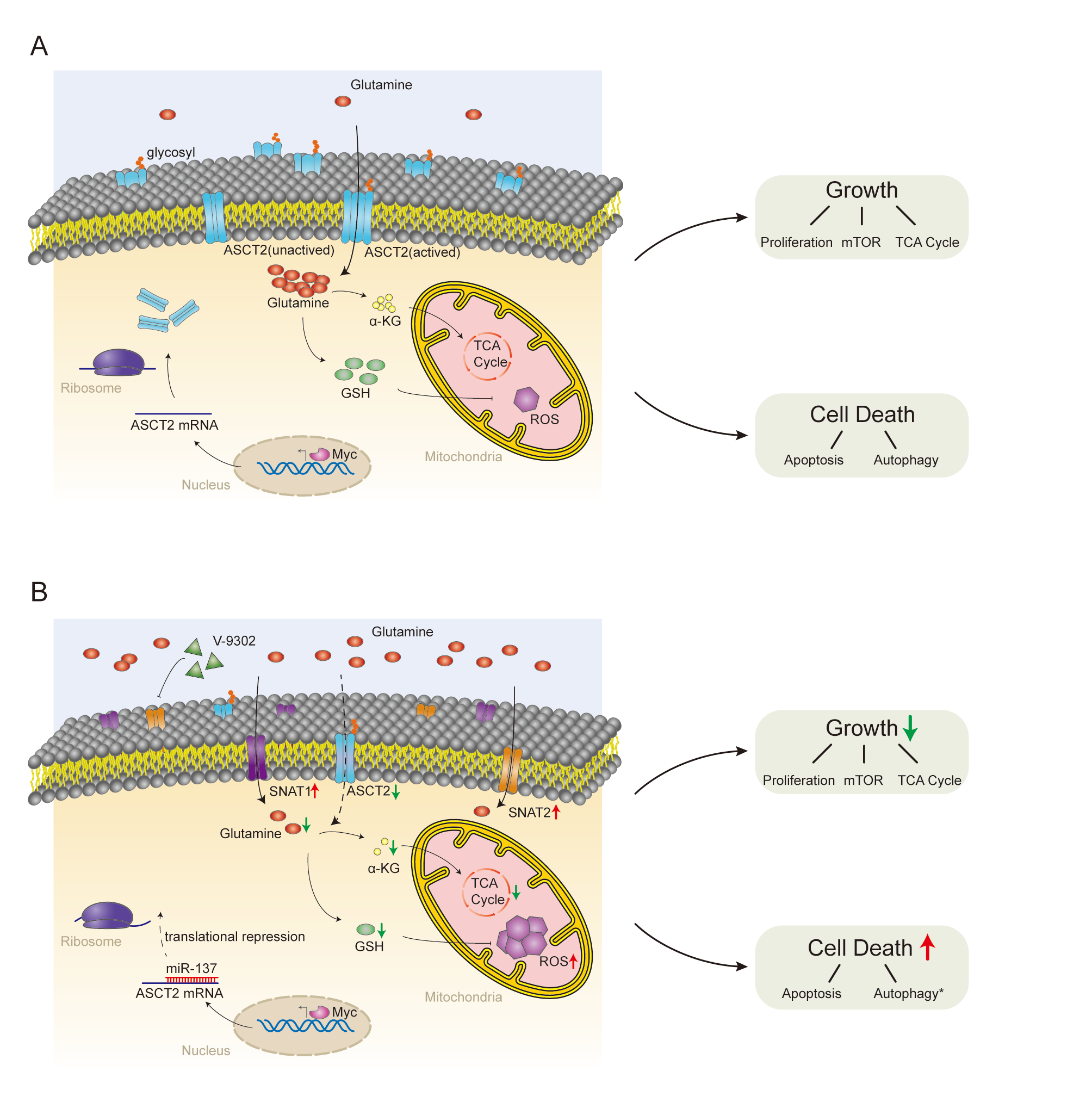


Supplementary Table 1 The correlation between ASCT2 expression and clinic pathological features in TCGA cohort.

| **Supplementary Table 1** The correlation between ASCT2 expression and clinic pathological features in TCGA cohort | | | |
| --- | --- | --- | --- |
|  | ASCT2 expression(mRNA) | | P value |
|  | low(n=250) | high(n=250) |  |
| **Age** |  |  | 0.28 |
| ≥60 | 104 | 116 |  |
| <60 | 146 | 134 |  |
| **Gender** |  |  | **0.006** |
| Female | 80 | 53 |  |
| Male | 170 | 197 |  |
| **Anatomic neoplasm subdivision** |  |  | **<0.001** |
| tonsil | 10 | 29 |  |
| Oral tongue | 81 | 45 |  |
| Larynx | 47 | 64 |  |
| Oral cavity | 42 | 30 |  |
| Floor of mouth | 23 | 37 |  |
| Alveolar ridge | 10 | 8 |  |
| Base of tongue | 9 | 14 |  |
| Buccal mucosa | 10 | 12 |  |
| Oropharynx | 5 | 4 |  |
| Hard palate | 4 | 3 |  |
| Hypopharynx | 6 | 4 |  |
| Lip | 3 | 0 |  |
| **Alcohol history** |  |  | 0.748 |
| No | 81 | 77 |  |
| Yes | 164 | 168 |  |
| **Tobacco smoking history** |  |  | 0.235 |
| No | 61 | 50 |  |
| Yes | 184 | 195 |  |
| **HPV** |  |  | **0.001** |
| Negative | 46 | 29 |  |
| Positive | 9 | 24 |  |
| **Lymphovascular invasion** |  |  | **0.017** |
| Absent | 119 | 100 |  |
| Present | 49 | 71 |  |
| **Neoplasm histologic grade** |  |  | **0.002** |
| Gx | 5 | 11 |  |
| G1 | 41 | 20 |  |
| G2 | 155 | 144 |  |
| G3 | 47 | 72 |  |
| G4 | 0 | 2 |  |
| **New tumor** |  |  | 1 |
| No | 226 | 226 |  |
| Yes | 24 | 24 |  |
| **Perineural invasion** |  |  | 0.317 |
| Absent | 87 | 99 |  |
| Present | 86 | 79 |  |
| **Pathological nodal extracapsular** |  |  | 0.659 |
| No extranodal extension | 112 | 114 |  |
| Microscopic extension | 36 | 39 |  |
| Gross extension | 16 | 20 |  |
| **pT stage** |  |  | 0.344 |
| pT1 | 28 | 17 |  |
| pT2 | 63 | 69 |  |
| pT3 | 51 | 45 |  |
| pT4 | 78 | 82 |  |
| **pN stage** |  |  | 0.114 |
| pN1 | 97 | 74 |  |
| pN2 | 32 | 33 |  |
| pN3 | 76 | 76 |  |
| pN4 | 1 | 6 |  |
| HPV: human papillomavirus | | | |

Supplementary Table 2 Gene sets enriched in patients with high ASCT2 expression (the TCGA cohort was divided into two groups: the top 25% with high ASCT2 expression and the bottom 25% with low ASCT2 expression) (h.all.v6.2.gmt).

| **Table S2** Gene sets enriched in patients with high ASCT2 expression(TCGA cohort was divided into two groups (top 25% with high ASCT2 expression and bottom 25% with low ASCT2 expression) ( h.all.v6.2.gmt) | | | |
| --- | --- | --- | --- |
| Gene sets | NES | NOM p-val | FDR q-val |
| HALLMARK_REACTIVE_OXIGEN_SPECIES_PATHWAY | 2.07 | 0.002 | 0.018 |
| HALLMARK_ADIPOGENESIS | 2.01 | <0.001 | 0.023 |
| HALLMARK_MYC_TARGETS_V1 | 2.00 | 0.006 | 0.017 |
| HALLMARK_E2F_TARGETS | 1.97 | 0.004 | 0.017 |
| HALLMARK_DNA_REPAIR | 1.95 | 0.002 | 0.018 |
| HALLMARK_OXIDATIVE_PHOSPHORYLATION | 1.94 | 0.008 | 0.015 |
| HALLMARK_G2M_CHECKPOINT | 1.94 | 0.008 | 0.013 |
| HALLMARK_MTORC1_SIGNALING | 1.91 | 0.014 | 0.015 |
| NES: Normalized Enrichment Score；NOM: Nominal; FDR: False Discovery Rate | | | |

Supplementary Table 3 Gene sets enriched in patients with high ASCT2 expression (the TCGA cohort was divided into two groups: the top 25% with high ASCT2 expression and the bottom 25% with low ASCT2 expression) (c2.cp.kegg.v6.2.symbols.gmt).

| **Table S3** Gene sets enriched in patients with high ASCT2 expression(TCGA cohort was divided into two groups (top 25% with high ASCT2 expression and bottom 25% with low ASCT2 expression) (c2.cp.kegg.v6.2.symbols.gmt) | | | |
| --- | --- | --- | --- |
| Gene sets | NES | NOM p-val | FDR q-val |
| KEGG_HUNTINGTONS_DISEASE | 2.01 | 0.004 | 0.052 |
| KEGG_BASE_EXCISION_REPAIR | 2.01 | <0.001 | 0.029 |
| KEGG_DNA_REPLICATION | 2.00 | <0.001 | 0.020 |
| KEGG_CELL_CYCLE | 2.00 | 0.002 | 0.016 |
| KEGG_GLUTATHIONE_METABOLISM | 1.99 | <0.001 | 0.014 |
| KEGG_MISMATCH_REPAIR | 1.99 | <0.001 | 0.013 |
| KEGG_SPLICEOSOME | 1.97 | 0.002 | 0.014 |
| KEGG_PYRIMIDINE_METABOLISM | 1.97 | 0.002 | 0.013 |
| KEGG_PORPHYRIN_AND_CHLOROPHYLL_METABOLISM | 1.94 | 0.002 | 0.018 |
| KEGG_HOMOLOGOUS_RECOMBINATION | 1.93 | 0.002 | 0.022 |
| NES: Normalized Enrichment Score; NOM: Nominal; FDR: False Discovery Rate | | | |
